# Association of Human Papillomavirus Type 16 Long Control Region Variations with Cervical Cancer in a Han Chinese Population

**DOI:** 10.7150/ijms.43030

**Published:** 2020-03-26

**Authors:** Shuying Dai, Chuanyin Li, Zhiling Yan, Ziyun Zhou, Xia Wang, Jun Wang, Le Sun, Li Shi, Yufeng Yao

**Affiliations:** 1 Institute of Medical Biology, Chinese Academy of Medical Sciences & Peking Union Medical College, Kunming 650118, China.; 2 School of Basic Medical Science, Kunming Medical University, Kunming 650500, China.; 3 Department of Gynaecologic Oncology, The 3rd Affiliated Hospital of Kunming Medical University, Kunming 650118, China.

**Keywords:** Human papillomavirus type 16, LCR, Variations, Cervical cancer, Association

## Abstract

**Objective**: High-risk human papillomavirus (HPV) E6 and E7 proteins are the major oncoproteins involved in the tumorigenesis of cervical cancer. The long control region (LCR) in HPV plays an important role in regulating the expression of the *E6* and *E7* oncogenes. In the current study, we investigated the association of HPV16 LCR variations with cervical cancer.

**Methods**: A total of 139 HPV16-positive cervical cancer patients (case group) and 116 HPV16-positive asymptomatic individuals (control group) were enrolled in the current study. Then, the HPV16 LCR was sequenced to determine the association between LCR variations and cervical cancer.

**Results**: In the current study, HPV16 A1-A3 (19.4%), A4 (78.4%) and D3 (2.2%) variants were found in the case group. However, only A1-A3 (34.5%) and A4 variants (65.5%) were found in the control group. The distribution of the HPV16 variants between the case and control groups was significantly different (*P*=0.009). Moreover, a total of eleven variations (A7167G, A7173C, C7176T, C7200T, T7269C, C7286A, C7729A, C7763T, A7841G, G7867A and T24C) were significantly different between the case and control groups (*P*<0.05). For the sub-lineage analysis, only C7873G variations were significantly different between the case and control groups in the A4 (As) variant (*P*=0.039).

**Conclusion**: Our results showed that specific variations in the HPV16 LCR were associated with cervical cancer. Our study will provide a good reference for further understanding of the relationship between HPV16 LCR variation and cervical cancer.

## Introduction

Human papillomavirus (HPV) plays an important role in cervical cancer 1. HPVs are subdivided according to their oncogenic potential into low-risk and high-risk types 2. Approximately 15 HPV genotypes (*i.e.* HPV16, 18, 31, 33, 35, 39, 45, 51, 52, 56, 58, 59, 68, 73, and 82) are recognized as high-risk types, according to an epidemiological survey 3,4, and each type of HPV acts as an independent infection with differing carcinogenic risks 4. Among the high-risk types, HPV16 is the most common type detected in patients with cervical cancer 5,6.

The HPV genome can be divided into three domains: a long control region (LCR) that contains sequences that control viral transcription and replication, an early region with open reading frames (ORFs) encoding the*E1*, *E2*, *E4*, *E5*, *E6* and *E7* genes, and a late region encoding two genes, *L1* and *L2* 2,7. The LCR, positioned between the end of the *L1* gene and the start of the *E6* gene, contains the early p97 promoter, which is responsible for the transcription of *E6* and *E7* genes and viral DNA replication 8. Cellular factors play important roles in stimulating and inhibiting p97 promoter activity, such as Oct-1, TEF-1 and YY1. In addition, the viral E2 protein can bind as a dimer to its palindromic recognition sites in the LCR to regulate the expression of *E6* and *E7* genes 8-14. The two major viral oncoproteins E6 and E7 play indispensable roles in promoting malignant transformation by targeting the p53 and retinoblastoma protein (pRB) tumour suppressors, respectively 15-18.

HPV16 has coevolved with human kind, and its variants segregate robustly into a phylogenetic tree with four phylogenetic lineages (A, B, C and D) and 16 sub-lineages: A1-A3 variant (previously known as EUR), A4 variant (As), B1-B4 variant (AFR1), C1-C4 variant (AFR2), D1 variant (NA), D2 variant (AA2), D3 variant (AA1) and D4 variant 19,20. Many studies reported that the carcinogenic capacity is different between sub-lineages, and the non-European lineages have stronger pathogenicity in developing cervix cancer in comparison with European lineages, such as the D3 variant (AA1) and A4 variant (As) 19,21. In addition to the HPV16 variants, viral genetic variations could play a vital role in cervical carcinogenesis 14,22-25. For example, the T variation of the E6 gene T350G (L83V) persisted more often than the G variation in the EUR sub-lineage 22.

Our previous study also showed that the HPV16 variants and the variations in the*E1*, *E2*, *E6* and *E7* genes were associated with cervical cancer in the Chinese Han population 26-28.Thus, in the current study, we investigated the distribution of LCR variation in the HPV16-positive asymptomatic individuals and HPV16-positive cervical cancer patients and found an association between the LCR variations and cervical cancer in a Han Chinese population.

## Materials and methods

### Ethical statement

All procedures were in accordance with the ethical standards of the responsible committee on human experimentation and with the Helsinki Declaration of 1964, which was revised in 2013. All experimental protocols were approved by the Institutional Review Boards of the No. 3 Affiliated Hospitals of Kunming Medical University. All participants provided written informed consent.

### Subjects

In the current study, the case group included 139 HPV16-positive cervical cancer patients, and 116 HPV16-positive asymptomatic individuals undergoing routine health check-ups were recruited as the control group. All individuals were recruited at the 3rd Affiliated Hospital of Kunming Medical University from 2017 to 2019. The sample type analysed inthe case group was cervical cancer tissues, and the sample type analysedin the control group was cervical exfoliated cells. The inclusion criteria: the patients were diagnosed according to “Diagnosis and Treatment Obstetrics and Gynaecology” and International Federation of Gynaecology and Obstetrics, 2009; the patients were not suffering from other malignancies; and the patients had not received chemotherapy and radiotherapy. The exclusion criteria were as follows: patients with prior malignant tumour history except cervical cancer; patients receiving radiotherapy or chemotherapy; and patients with an unclear pathological diagnosis. Over the same period, the healthy HPV16-positive asymptomatic women undergoing the healthy routine screen at the same hospital served as the healthy controls in the current study. All participants were self-reported as ethnically Han in the current study.

### HPV genotyping

For HPV genotyping, the Tellgenplex™ HPV DNA Test, which is a suspension bead array method for identifying HPV types, was used. The experimental protocol has been described in a previous study 29. Human β-globin was used as an internal control for each reaction.

### Amplification and sequencing of the HPV16 LCR gene

The HPV16 LCR was amplified using a high-fidelity DNA polymerase with the primers 5'-TACCTCTACAACTGCTAAACGC-3' and 5'-CCTGTGGGTCCTGAAACA-3' (912 bp); the PCR product contained the complete LCR sequence, with a length of 832 bp, and the reference sequence used was AF534061.1. PCR was performed in a final volume of 25 μl, and each PCR mixture contained 1× Q5 PCR buffer (containing Mg2+), 200 μM dNTPs, 0.5 μM sense and antisense primers, 50 ngof genomic DNA, and 0.02 U/μl high-fidelity DNA polymerase. The PCR conditions were as follows: an initial denaturation step at 98°C for 3 min, 35 cycles of denaturation at 98°C for 10 s, annealing at 60°C for 30 s, and extension at 72°C for 90 s; and a final elongation at 72°C for 2 min. The amplified DNA fragments (including the complete LCR sequence) were sent to Shanghai Sangon Biotech for sequencing after visualization with a 1.0% agarose electrophoresis assay. The primers used for LCR sequencing were 5'-AATCCCTGTTTTCCTGACCTG-3', 5'-CAAGCCAAAAATATGTGCCTAAC-3'.

### Identification and phylogenetic analyses of HPV16 LCR variations

All sequences of the HPV16 LCR sequences were assembled by SeqMan software and aligned by the ClustalW multiple sequence alignment tool included in the Molecular Evolutionary Genetics Analysis (MEGA) v7.0 software package. The HPV16 reference sequence AF534061.1 was used to identify variations in the HPV16 LCR sequences. Phylogenetic trees of the HPV16 LCR were constructed using MEGA 7.0 software by the maximum likelihood method by bootstrapping with 1000 replications. The reference sequences used to construct the phylogenetic tree were obtained from GenBank, and the accession numbers are HQ644283.1 (A1), HQ644268.1 (A1), HQ644280.1 (A1), HQ644282.1 (A1), AF536179.1 (A2), HQ644236.1 (A3), HQ644248.1 (A4), HQ644251.1 (A4), AF534061.1 (A4), HQ644235.1 (A4), HQ644240.1 (B1), HQ644290.1 (B1), HQ644238.1 (B1), HQ644298.1 (B3), HQ644237.1 (C), HQ644239.1 (C), HQ644249.1 (C), HQ644250.1 (C), AF472509.1 (C), HQ644257.1 (D1), HQ644279.1 (D2), HQ644281.1 (D2), HQ644263.1 (D2), HQ644277.1 (D2), HQ644247.1 (D3), HQ644253.1 (D3), HQ644255.1 (D3), and AF402678.1 (D3).

### Statistical analysis

The frequency of each HPV16 LCR variation was determined by direct counting. A chi-square test, Fisher's exact test or continuity correction test was performed to analyse the associations of HPV16 LCR variations with cervical cancer. The statistical analyses were performed using SPSS 13 (Chicago, IL), and* P* values less than 0.05 were considered statistically significant.

## Results

### Distribution of HPV16 variants in the case and control groups

**Figure 1:**
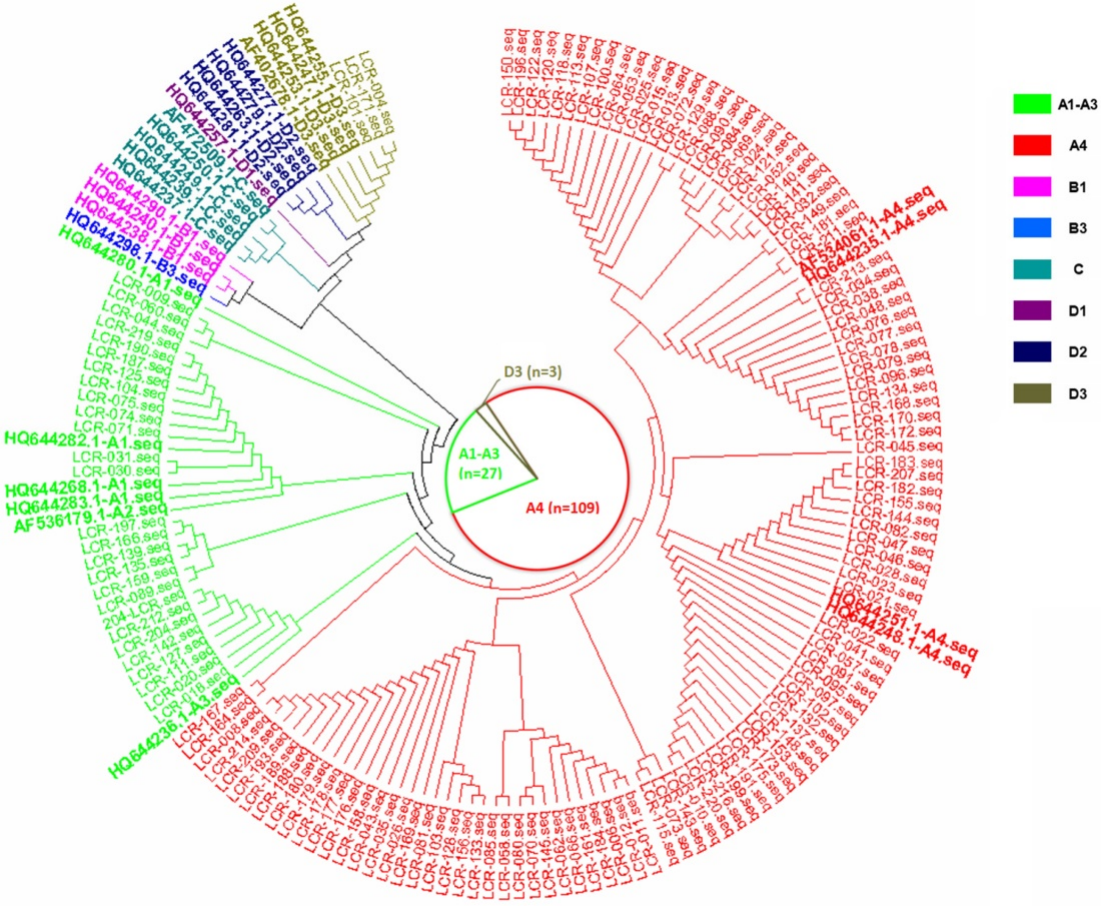
The phylogenetic tree based on the HPV16 LCR sequences from HPV16-positive cervical cancer patients, corresponding to the AF534061.1 reference sequence (nucleotide positions 7156-7905, 1-82).

**Figure 2:**
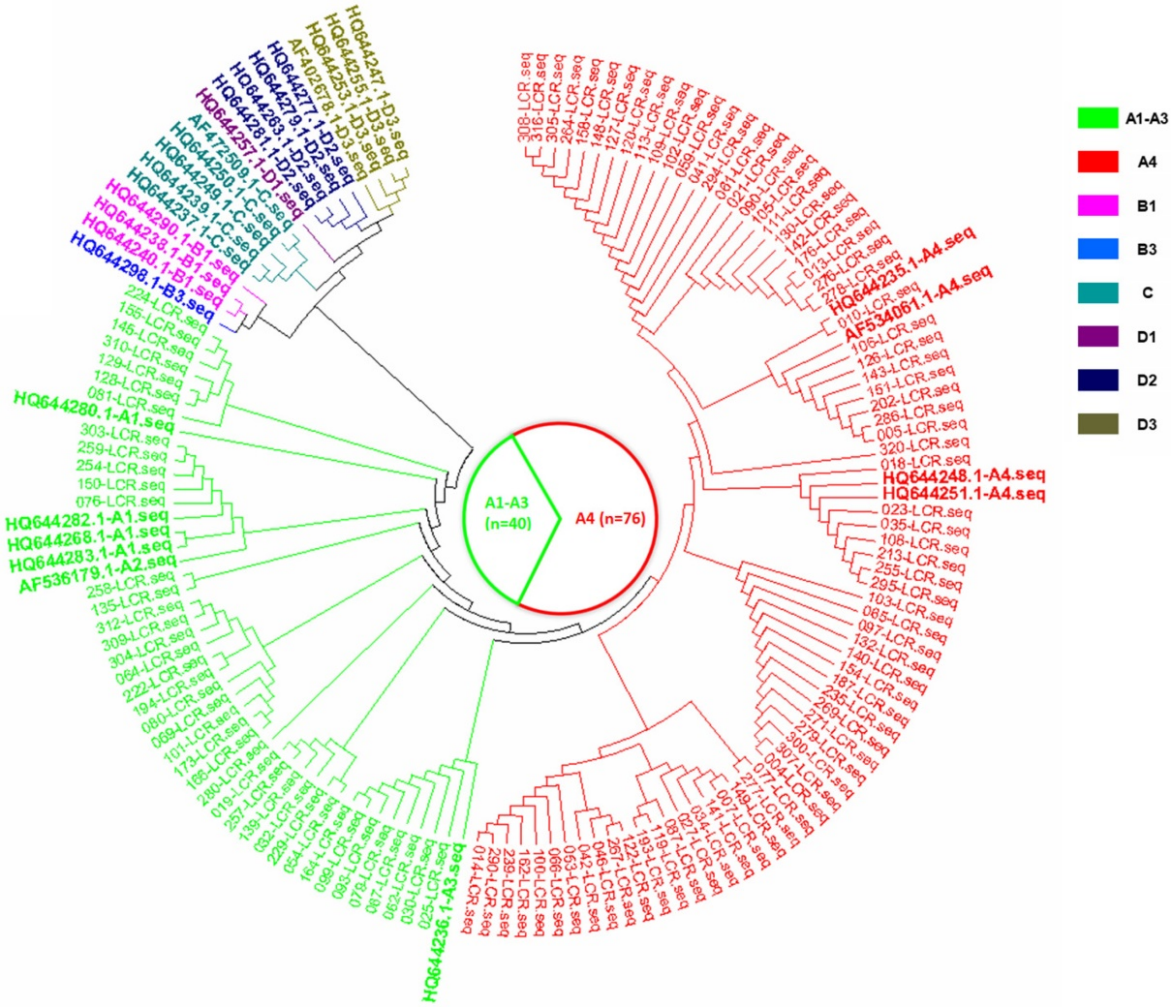
The phylogenetic tree based on the HPV16 LCR sequences from HPV16-positive asymptomatic individuals, corresponding to the AF534061.1 reference sequence (nucleotide positions 7156-7905, 1-82).

In the current study, there were three HPV16 sub-lineages in the case group, namely, A1-A3 (EUR), A4 (As) and D3 (AA1), with distribution frequencies of 19.4%, 78.4% and 2.2%, respectively (Figure 1). In the control group, only the A1-A3 (EUR) and A4 (As) sub-lineages were found, with distribution frequencies of 34.5% and 65.5%, respectively (Figure 2). The difference in the distribution of the HPV16 variants between the case group and control group was statistically significant (*P*=0.009).

### HPV16 LCR variations in the case and control groups

The HPV16 LCR variations in the case and control groups are shown in Table 1 (Reference sequence AF534061.1). Thirty-nine variations were observed in the case group, and thirty-two variations were observed in the control group. Among these variations, a total of eleven variations (A7167G, A7173C, C7176T, C7200T, T7269C, C7286A, C7729A, C7763T, A7841G, G7867A and T24C) were statistically significant between the case group and control group (*P*<0.05).

### Distribution of HPV16 LCR gene variations in the A4 (As), A1-A3 (EUR) and D3 (AA1) sub-lineages

Table 2 shows HPV16 LCR gene variations in the A1-A3 (EUR), A4 (As) and D3 (AA1) sub-lineages. A total of eighteen and twenty-three variations were found in the case and control group in the A1-A3 (EUR) variants, respectively, and no variations were significantly different between the case and control group (*P*>0.05). However, A7173C showed a trend to associate with cervical cancer (*P*=0.074) in the A1-A3 (EUR) variants (case: 0% vs control: 15%). In the HPV16 A4 (As) variant, fifteen and thirteen variations were found in the case and control groups, respectively. The C7873G variation was significantly different between the case (8.3%) and control (18.4%) group (*P*=0.039), and the A7232C showed a trend to associate with cervical cancer (case: 0% vs control: 3.9%; *P*=0.068) in the HPV16 A4 (As) variant.

## Discussion

The HPV16 LCR contains the transcriptional enhancer and promoter regions as well as the origin of DNA replication. The LCR variations can have various transcriptional activities by changing the activity of the promoter 8-14. In the current study, we investigated the distribution of LCR variation in HPV16-positive asymptomatic individuals and HPV16-positive cervical cancer patients. Our results showed that the distribution of the HPV16 variants and variations in LCR were associated with the development of cervical cancer.

Previous studies have indicated that HPV16 variants have different carcinogenic potentials 30-32. In the current study, we found that the frequencies of the A4 (As) variant were significantly different between the case and control groups (78.4% vs 65.5%, respectively). We also found that the D3 (AA1) variant was only found in the case group (2.2%). These results were concordant with our previous finding and other studies showing that non-A1-A3 (non-EUR), such as A4 (As) and D3 (AA1) variants, could have a higher risk of progression to cervical cancer than A1-A3 (EUR) variants 14,26,28,33-36. The reason for these differences could be that HPV intratypic variants have various transcriptional activities that alter biological and biochemical properties and lead to differences in the pathogenicity of these variants 37.

Previous studies have reported that HPV-16 LCR E2 binding sites serve as landmarks to divide the LCR into functionally distinct segments, which have been called the 5', the central, and the 3′ segment of LCR 12. The 5' segment of the HPV16 LCR has a size of approximately 300 bp and is bracketed by the termination codon of L1 and the first E2 binding site (7455 site) 12. The 5' segment of HPV16 LCR contains a transcription termination site and a negative regulatory element acting at the level of late mRNA stability 36,48. In the current study, the distributions of A7167G and A7173C were located in the 5' segment of the LCR of HPV16, and our results showed that the distributions of the two variations were significantly different between the case and control groups (*P*=0.007 and *P*=0.008, respectively). Connor *et al*. and Xi *et al*. reported that A7167G and A7173C are located in the binding site of the transcription factor AP1 and FOXA1 12,13. FOXA1 acts as a key transcription factor to regulate the progression of cancer in different cancers, such as breast, liver and lung cancer 38-42. For AP1, one of the AP1 roles could translate increased mitogenic signals into increased levels of viral gene transcription through E5 action 43. The other role could be that AP1 mediates the repressive effect of retinoids, which are the molecular antagonists to AP1 44, on HPV gene expression 45. Thus, these two variations could change the binding of transcription factors to play an important role in *E6* and *E7*gene expression.

The central segment of the HPV16 LCR is flanked by two E2 protein binding sites (7455and 7862), which have a size of approximately 400 bp 12. The central segment of the HPV16 LCR is an epithelial-specific transcriptional enhancer region that has many transcription factor binding sites and plays an important role in the regulation of the gene expression of*E6* and *E7* 46,47. In the current study, we found that the frequencies of C7729A, C7763T and A7841G, which are located in the enhancer region, showed significant differences between the case and control groups (*P*=0.033, 0.017, and 0.022, respectively). C7729A is located in the enhancer region and proximal to the binding site of YY1, NF1, Tef-1 and Octamer factor-1 transcription factors 8,12,14. Although C7763T and A7841G were not located in the enhancer region, these two sites are close to the enhancer region and the binding sites of NF1 and YY1, respectively 8,12,14. Several studies have reported that YY1, NFI, TEF-1 and Octamer factor-1 have roles in viral replication, regulate viral and cellular genes and influence enhancer activity 48-52. Thus, these variations could change the binding of transcription factors to influence *E6* and *E7* gene expression.

The 3' segment of the HPV16 LCR was between the second E2 protein binding site (7862) and the *E6* gene and has a size of approximately 140 bp 12. Several studies have reported that variations in the p97 promoter, which is located in the *E6* gene proximal part of the LCR, could influence the transcription of *E6* and *E7* genes. In the current study, we found that the distribution of T24C showed a significant difference between the control and case groups (*P*=0.043), which indicated that this variation could induce dysregulation of *E6* and *E7* gene expression through changing the cellular and viral transcription factor binding sites. In 2013, Pientong *et al.* found that G7428A and C7873G in the A4 (As) variant, which are proximal to the p97 promoter, showed higher transcriptional activity than other A4 (As) and A1-A3 (EUR) variants 14. In the current study, our results also showed that the distribution of C7873G was significantly different between the case and control groups in the A4 (As) variant (*P*=0.039). These results indicated that the oncogenic potential of the A4 (As) variant could be influenced by variations that are proximal to the promoter region.

The viral E2 protein, which binds as a dimer to LCR recognition sites, is considered a negative regulator by repressing or activating the expression of *E6* and *E7* genes 53. In the current study, G7867A variation, which is likely one of the binding sites of E2 protein, was only found in the control group. This variation could play a role in the occurrence of cervical cancer by affecting the binding efficiency of the HPV16 E2 protein.

One of the limitations in the current study was that our data only presented the case-control study results and there was a lack of functional validation that would allow for a closer understanding of the relationship between intratypic variation and the carcinogenic potential of the most frequent high-risk HPV genotypes. In the future, we will do the variations function validation to find the role of the viral genetic variability in the different courses of HPV-associated diseases.

In the current study, our results showed that the distribution of the HPV16 variants was significantly different between the case group and control groups, and D3 (AA1) was only found in the case group. In addition, we also found that HPV16 LCR variations (A7167G, A7173C, C7176T, C7200T, T7269C, C7286A, C7729A, C7763T, A7841G, G7867A and T24C) were statistically significant between the case group and control groups. Moreover, the distribution of C7873G variation was significantly different between the case and control groups in the A4 (As) variant, and no variations were significantly different between the case and control groups in the A1-A3 (EUR) variant.

## Figures and Tables

**Table 1:** HPV16 LCR variations in the case and control groups

HPV16 LCR segment	HPV16 Genome position	Case (*n*=139)		Control (*n*=116)		*P* Value
		Variation	Frequency (%)	Variation	Frequency (%)	
5' segment	A7167G	14	10.1	26	22.4	0.007^a^
	A7173C	0	0.0	6	5.2	0.008^c^
	C7174A	33	23.7	40	34.5	0.059^a^
	T7175G	2	1.4	2	1.7	1.000^c^
	C7176T	30	21.6	40	34.5	0.022^a^
	C7200T	30	21.6	40	34.5	0.022^a^
	A7232C	4	2.9	8	6.9	0.131^a^
	T7269C	30	21.6	40	34.5	0.022^a^
	C7286A	30	21.6	40	34.5	0.022^a^
	C7288A	70	50.4	61	52.6	0.723^a^
	T7301G	2	1.4	0	0.0	0.502^c^
	C7309T	2	1.4	5	4.3	0.251^c^
	A7338T	3	2.2	0	0.0	0.253^c^
	T7379C	0	0.0	2	1.7	0.206^c^
	C7393T	3	2.2	2	1.7	1.000^c^
	C7394T	5	3.6	5	4.3	1.000^c^
	A7418G	18	12.9	14	12.1	0.833^a^
	G7428A	26	18.7	25	21.6	0.571^a^
	G7435T	2	1.4	0	0.0	0.502^c^
	A7451C	2	1.4	0	0.0	0.502^c^
central segment	A7484C	3	2.2	0	0.0	0.253^c^
	G7488A	3	2.2	0	0.0	0.253^c^
	C7613A	3	2.2	1	0.9	0.622^c^
	A7659G	15	10.8	5	4.3	0.055^a^
	C7668T	3	2.2	0	0.0	0.253^c^
3' segment	C7688A	3	2.2	0	0.0	0.253^c^
	T7699C	0	0.0	2	1.7	0.206^c^
	G7701A	0	0.0	2	1.7	0.206^c^
	T7713G	3	2.2	7	6.0	0.206^b^
	A7728C	3	2.2	0	0.0	0.253^c^
	C7729A	22	15.8	31	26.7	0.033^a^
	T7742G	3	2.2	0	0.0	0.253^c^
	C7763T	7	5.0	0	0.0	0.017^c^
	C7780T	108	77.7	97	83.6	0.236^a^
	C7785T	3	2.2	0	0.0	0.253^c^
	G7825A	9	6.5	4	3.4	0.274^a^
	A7829C	12	8.6	7	6.0	0.431^a^
	A7841G	30	21.6	40	34.5	0.022^a^
	G7867A	0	0.0	4	3.4	0.042^c^
	A7872G	10	7.2	11	9.5	0.508^a^
	C7873G	9	6.5	14	12.1	0.120^a^
	C7885G	3	2.2	0	0.0	0.253^c^
E6 gene	C13T	6	4.3	2	1.7	0.298^c^
	T24C	32	23.0	40	34.5	0.043^a^
	A49C	0	0.0	2	1.7	0.206^c^

**Note:** The reference HPV16 *LCR* sequence used was AF534061.1.**^a^** Pearson Chi-Square; **^b^** Continuity Correction;**^c^** Fisher's Exact Test.

**Table 2:** HPV16LCR variations in the case and control groups in A1-A3, A4 and D3 sub-lineages

Genome position	A1-A3-Case (*n*=27)		A1-A3-Control (*n*=40)		*P* Value	A4-Case (*n*=109)		A4-Control (*n*=76)		*P* Value	D3-Case (*n*=3)	
	Variation	Frequency (%)	Variation	Frequency (%)		Variation	Frequency (%)	Variation	Frequency (%)		Variation	Frequency (%)
A7167G	14	51.9	26	65.0	0.282^a^	0	0.0	0	0.0	-	0	0.0
A7173C	0	0.0	6	15.0	0.074^c^	0	0.0	0	0.0	-	0	0.0
C7174A	27	100.0	40	100.0	-	3	2.8	0	0.0	0.270^c^	3	100.0
T7175G	2	7.4	2	5.0	1.000^c^	0	0.0	0	0.0	-	0	0.0
C7176T	27	100.0	40	100.0	-	0	0.0	0	0.0	-	3	100.0
C7200T	27	100.0	40	100.0	-	0	0.0	0	0.0	-	3	100.0
A7232C	1	3.7	5	12.5	0.197^c^	0	0.0	3	3.9	0.068^c^	3	100.0
T7269C	27	100.0	40	100.0	-	0	0.0	0	0.0	-	3	100.0
C7286A	27	100.0	40	100.0	-	0	0.0	0	0.0	-	3	100.0
C7288A	27	100.0	40	100.0	-	40	36.7	21	27.6	0.197^a^	3	100.0
T7301G	2	7.4	0	0.0	0.159^c^	0	0.0	0	0.0	-	0	0.0
C7309T	2	7.4	5	12.5	0.693^c^	0	0.0	0	0.0	-	0	0.0
A7338T	0	0.0	0	0.0	-	0	0.0	0	0.0	-	3	100.0
T7379C	0	0.0	2	5.0	0.512^c^	0	0.0	0	0.0	-	0	0.0
C7393T	0	0.0	2	5.0	0.512^c^	0	0.0	0	0.0	-	3	100.0
C7394T	2	7.4	5	12.5	0.693^c^	0	0.0	0	0.0	-	3	100.0
A7418G	0	0.0	0	0.0	-	18	16.5	14	18.4	0.736^a^	0	0.0
G7428A	0	0.0	0	0.0	-	26	23.9	25	32.9	0.176^a^	0	0.0
G7435T	0	0.0	0	0.0	-	2	1.8	0	0.0	0.513^c^	0	0.0
A7451C	2	7.4	0	0.0	0.159^c^	0	0.0	0	0.0	-	0	0.0
A7484C	0	0.0	0	0.0	-	0	0.0	0	0.0	-	3	100.0
G7488A	0	0.0	0	0.0	-	0	0.0	0	0.0	-	3	100.0
C7613A	0	0.0	0	0.0	-	3	2.8	1	1.3	0.645^c^	0	0.0
A7659G	0	0.0	0	0.0	-	15	13.8	5	6.6	0.122^a^	0	0.0
C7668T	0	0.0	0	0.0	-	0	0.0	0	0.0	-	3	100.0
C7688A	0	0.0	0	0.0	-	0	0.0	0	0.0	-	3	100.0
T7699C	0	0.0	2	5.0	0.512^c^	0	0.0	0	0.0	-	0	0.0
G7701A	0	0.0	2	5.0	0.512^c^	0	0.0	0	0.0	-	0	0.0
T7713G	3	11.1	7	17.5	0.711^b^	0	0.0	0	0.0	-	0	0.0
A7728C	0	0.0	0	0.0	-	0	0.0	0	0.0	-	3	100.0
C7729A	19	70.4	31	77.5	0.511^a^	0	0.0	0	0.0	-	3	100.0
T7742G	0	0.0	0	0.0	-	0	0.0	0	0.0	-	3	100.0
C7763T	0	0.0	0	0.0	-	4	3.7	0	0.0	0.145^c^	3	100.0
C7780T	27	100.0	40	100.0	-	78	71.6	57	75.0	0.604^a^	3	100.0
C7785T	0	0.0	0	0.0	-	0	0.0	0	0.0	-	3	100.0
G7825A	0	0.0	0	0.0	-	9	8.3	4	5.3	0.433^a^	0	0.0
A7829C	0	0.0	0	0.0	-	12	11.0	7	9.2	0.692^a^	0	0.0
A7841G	27	100.0	40	100.0	-	0	0.0	0	0.0	-	3	100.0
G7867A	0	0.0	3	7.5	0.267^c^	0	0.0	1	1.3	0.411^c^	0	0.0
A7872G	0	0.0	0	0.0	-	10	9.2	11	14.5	0.264^a^	0	0.0
C7873G	0	0.0	0	0.0	-	9	8.3	14	18.4	0.039^a^	0	0.0
C7885G	0	0.0	0	0.0	-	0	0.0	0	0.0	-	3	100.0
C13T	0	0.0	0	0.0	-	6	5.5	2	2.6	0.474^c^	0	0.0
T24C	27	100.0	40	100.0	-	2	1.8	0	0.0	0.513^c^	3	100.0
A49C	0	0.0	2	5.0	0.512^c^	0	0.0	0	0.0	-	0	0.0

**Note:** The reference HPV16 *LCR* sequence used was AF534061.1. **^a^** Pearson Chi-Square; **^b^** Continuity Correction; **^c^** Fisher's Exact Test.
